# Health Behaviors of a Sample of Adolescents in Bandar Abbas, Iran

**DOI:** 10.5812/ijhrba.8842

**Published:** 2013-06-26

**Authors:** Teamur Aghamolaei, Sedigheh Sadat Tavafian

**Affiliations:** 1 Department of Public Health, School of Health, Hormozgan University of Medical Sciences, Bandar Abbas, IR Iran; 2 Department of Health Education, Faculty of Medical Sciences, Tarbiat Modares University, Tehran, IR Iran

**Keywords:** Health Behavior, Adolescent, Iran

## Abstract

**Background:**

Health promotion for adolescents has become a research priority worldwide and life at school offers a good opportunity to establish health promoting behavior among this age group.

**Objectives:**

This study aimed to investigate health behaviors of a sample of adolescents in Bandar Abbas, Iran.

**Materials and Methods:**

Totally, 410 students including 204 males and 206 females studying in grades 9 to 12 and aged between 15–18 years old were studied. The instruments used to collect data were a self administered demographic questionnaire and the scale of Adolescent Health Promotion (AHP).

**Results:**

The mean age of participants was 16.5 (SD = 1.34) years. The mean score of Adolescent Health Promotion (AHP) scale was 64.8 (SD = 8.9) ranging from 34.3 to 89.9. Female students scored significantly higher for health-responsibility, life-appreciation, and stress-management than male students (P < 0.05). In contrast, male students scored higher scores on exercise behavior than female (P < 0.05). All dimensions of AHP scale except for nutrition behavior and social support was associated with gender and nutrition behavior was associated with age (P < 0.05).

**Conclusions:**

This study indicated that sedentary life style and physical inactivity is a common and serious problem among high school students of Bandar Abbas.

## 1. Background

There are an estimated 1.2 billion adolescents - one in every five people – living throughout the world. On the other hand, the vast majority of changes in physical, psychological, and social interactions happen during the adolescence period (1). Adolescents are usually thought of as a healthy group. Nevertheless, many adolescents do die prematurely due to preventable events resulting from risky behaviors like accidents, suicide, violence, pregnancy related complications due to unprotected sexual relationship, substance abuse and other illnesses (2). In many countries, adolescent health problems are considered as national health priorities (3). Various unhealthy behaviors such as smoking, alcohol use, and not using condom are usually initiated during the developmental periods of adolescence or childhood. Child and adolescent behavior may predict health behaviors and health status in early adulthood (3, 4). Youth is an opportunity to explore needs, make decisions and form social relationships, so habits established during this part of life would continue for the whole life (5). Nowadays, considering health needs of adolescents and promoting their health has become a research priority worldwide (6). Among healthy behaviors, healthy diet and regular physical activity are major factors influential on adolescent health promotion throughout their entire life. On the other hand, schools offer a good opportunity for establishment of these healthy behaviors among this age group (7, 8).

In London, a study on adolescents found that many overweight teenagers, especially boys, did not regularly realize that they were too fat (9). Another study showed that adolescents who believed on high weight controllability were more likely to express that thin individuals, compared to fat ones, would engage and benefit from physical activity and would be satisfied regarding their weight (10). Although international research has identified several socio-economic and cultural factors that might be associated with overweight children (11), few corresponding studies have been performed in eastern countries (12). A systematic review from Iran showed that the prevalence rate of mental disorders reported by two studies using diagnostic instruments was equal to 16.6% and 4.34 %, so there was a significant heterogeneity between the studies. This study concluded that the prevalence rates of mental disorders reported for high school students of Iran had a wide range so more studies with improved quality are needed in this field (13). A previous study conducted in Iran showed Iranian girls faced many barriers to an active lifestyle such as lack of enough places to exercise, access to facilities and resources, cultural limitations, and paid lower attention to exercise over other duties such as doing homework or home responsibilities. This study suggested that access to suitable facilities and a supportive environment are important strategies for promoting physical activity among female adolescents in this country (14). To the best of our knowledge, researches regarding adolescents’ health behaviors in Iran especially in Bandar Abbas – a city in the south of Iran - are very rare, so there is no doubt that this study is the first preliminary research to investigate the status of health behaviors among adolescents living in this area. These data provided a first step towards the foundation of knowledge necessary to develop interventional preventive programs to improve health promoting behaviors of these adolescents. With this regard, this study aimed to assess health behaviors of a sample of adolescents in Bandar Abbas, Iran.

## 2. Objectives

This study aimed to investigate health behaviors of a sample of adolescents in Bandar Abbas, Iran.

## 3. Materials and Methods

This was a cross-sectional study conducted for high school students of Bandar Abbas. Written consent forms were signed by the adolescents before entering the study.

The target population of this study included adolescents who were studying in the high schools of Bandar Abbas. This city which is located in the south of Iran, has 58 female and 67 male high schools in which students in grade 9, 10 and 11 study. In Iran, students in grade 12 study in pre-college centers. At the time of the study, there were 20 female and 17 male pre-college centers in Bandar Abbas that were located in different geographical points of the city. For the first step of sampling, 20 sites (including five female high schools, five female pre-college centers, five male high schools and five male pre-college centers) were selected randomly. For the second step, in each high school 33 students (11 students from each grade) and in each pre-college center 11 students (grade 12) were recruited if they agreed to take part in the study. In total, 440 students were recruited. Thirty students filled out the questionnaires incompletely. Thus, a total of 410 questionnaires (204 male and 206 female students) were analyzed. The response rate was 93.2%.

In this study a self-administered questionnaire regarding demographic characteristics, and the scale of Adolescent Health Promotion (AHP) were used as data collection instruments. Demographic characteristics included age, sex, and grade. The Adolescent Health Promotion (AHP) scale assessed health-promoting behaviors. The AHP scale was designed based on the literature and guided by the theoretical framework of Pender’s health promotion model (15). This model was derived from Bandura's Social Cognitive Theory (16). Moreover, this scale has been used by Iranian studies in which physical activity among adolescents were assessed (14, 17). The AHP scale consisted of a set of 40 items that assessed six dimensions of healthy behavior including nutrition, social support, life appreciation, health responsibility, stress management, and exercise. The frequency of reported behaviors was obtained using a self-reporting Likert scale with a five-point response format, "never, rarely, sometimes, usually, always", with the rating score ranging from 1 to 5. Nutrition consisted of six items scored from 6 to 30, social support consisted of seven items scored from 7 to 35, life appreciation consisted of eight items scored from 8 to 40, health responsibility consisted of eight items scored from 8 to 40, stress management consisted of seven items scored from 7 to 35, and exercise behavior consisted of four items scored from 4 to 20. Then, raw scores for each of the six AHP dimensions were converted to a value for the dimension from 0 to 100 according to the Wang et al. procedure (6). Higher scores indicated higher health promoting behavior. A panel of experts evaluated content validity and a pilot test was performed to determine whether it would be appropriate for the target population of this study. This scale was applied in a similar study on adolescents of Turkey (18). The reliability coefficient for each dimension that was calculated using Cronbach’s alpha coefficients for nutrition, social support, life appreciation, health responsibility, stress management, and exercise behavior, were calculated as 0.66, 0.70, 0.88, 0.72, 0.76 and 0.85 respectively. Following clear instructions and clarifying the aim of the study, the questionnaires were distributed to the students. Data Analysis: Descriptive statistics and student t-test were used to analyze the data by the SPSS software version 16 and P < 0.05 was considered statistically significant

## 4. Results

The mean age of participants was 16.5 (SD = 1.34) years, ranging from 15 to 18. The majority of participants (50.2%) were male. Table 1, shows the demographic characteristics of the participants.

**Table 1.** Demographic Characteristics of the Subjects

	No. (%)
**Age ,years, (Mean ± SD)**	
15 (16.5 ± 1.34)	99 (24.1)
16 (16.5 ± 1.34)	94 (22.9)
17 (16.5 ± 1.34)	112 (27.3)
18 (16.5 ± 1.34)	105 (25.6)
**Gender**	
Male	206 (50.2)
Female	204 (49.8)
**Grade**	
9^th^	104 (25.4)
10^th^	120 (29.3)
11^th^	109 (26.6)
12^th^	77 (18.8)
**Fathers’ education**	
Illiterate	12 (2.9)
Primary school	45 (11)
Secondary school	103 (25.1)
High school	161 (39.3)
University	89 (21.7)
**Mothers’ education**	
Illiterate	38 (9.3)
Primary school	96 (23.4)
Secondary school	91 (22.2)
High school	116 (28.3)
University	69 (16.8)

The mean score on the AHP scale was 64.8 (SD = 8.9), with a range of 34.3 to 89.9. Table 2 compares the mean scores of all the behaviours between the two genders. There were significant differences between male and female students in terms of health-responsibility, life-appreciation, exercise and stress-management dimensions (P < 0.05). Female students scored slightly higher on health-responsibility, life-appreciation, and stress-management and the differences were statistically significant (P < 0.05). In contrast, male students scored higher than female students on exercise behaviour. This difference was statistically significant (P < 0.05). However, there was no significant difference between male and female students in terms of nutrition behaviour and social support dimensions.

**Table 2.** Comparison of Health Behavior Scores Between Male and Female Students (T-test)

	Male, Mean ± SD, (N = 206)	Female, Mean ± SD, (N = 204)	Total, Mean ± SD	P Value^a^
**Nutrition behavior**	72. ± 12.1	70.07 ± 14.3	71.06 ± 13.2	0.13
**Social support**	62.10 ± 12.1	63.91 ± 14.9	63.00 ± 13.6	0.17
**Health responsibility**	61.25 ± 15.2	65.01 ± 14.6	63.12 ± 15.1	0.01
**Life appreciation**	80.26 ± 14.4	83.97 ± 13.4	82.11 ± 14.1	0.007
**Exercise behavior**	49.81 ± 23.7	33.08 ± 25.1	41.49 ± 25.8	0.000
**Stress management**	66.67 ± 12.8	70.51 ± 14.2	68.58 ± 13.7	0.004

^a^Significant

## 5. Discussion

Although, in recent years, research on risky behaviors among Iranian adolescents was given more attention, assessing health-promoting behaviors of this age group especially through the AHP scale, was not yet performed. This study extended our knowledge about the health promoting behaviors of high school students living in a southern city of Iran. According to this study, the mean scores of most dimensions of AHP were lower than 70. Particularly the mean score of exercise behavior was the lowest. This result indicated that sedentary life style and physical inactivity is a common and serious problem among high school students studied in this research. A previous study conducted in Iran (19), stated that three national surveys conducted among Iranian adults have shown more than 80% of the Iranian population are physically inactive. Furthermore, another study (20), indicated that Iranian youth have a sedentary life style that might be due to spending too much time watching television and playing computer games, as well as due to decreased opportunities for exercise in schools and communities. The results of the present study are consistent with the findings of previous investigations regarding the mean score of exercise behavior being the lowest among adolescents (6, 21). In addition to this finding about low rate of adolescents’ exercise behavior, the results of this study showed that female students had lower exercise behaviors than male students. This finding is similar to the results of other studies that reported adolescent girls had lower levels of physical activity compared to their male counterparts (6, 22, 23). Our study also showed that health responsibility and social support scores were the second lowest among all dimensions of AHP scale. What we can rationale about feeling low social support and health responsibility of adolescents living in Bandar Abbas, is that the majority of these adolescents live with low or uneducated parents. Chen and co- workers revealed in their study (12), that living with less educated parents was associated with lower health promoting behaviors. This result is also consistent with what was reported by Wang and co-workers (6), with this difference that they examined health-promoting behaviors among university students. Furthermore, Huurre and co-workers concluded in their study (24) that parental socio demographic status has an influence on early adult health behavior. As this study found, the mean scores of stress management and nutrition behavior were near 70. Since there is no data regarding these behaviors among representative Iranian adolescents, there is no possibility to compare the status of stress management and nutrition behavior of high school students of Bandar Abbas with the entire population of adolescents in Iran. However, previous research on mental health of Iranian adolescents showed no satisfactory position for adolescents in this regard (24, 25). As we can see in this study, the mean score of life-appreciation was the highest. This indicated that adolescents living in Bandar Abbas were satisfied with their life. The rationale that could be argued for this finding is that the adolescents studied in this study live in one of the most disadvantaged provinces of Iran and they probability have low expectation of life. In conclusion, this study provided basic data about health promoting behavior of this target group and doing more research in this regard is warranted. This study provided evidence for gender differences in all dimensions of AHP scale except for nutrition behavior and social support. Female students were more likely to take health responsibility, to appreciate their life and to manage their stress. However, male students were more likely to engage in exercise. A previous study conducted among university students of China reported that male students did more exercise than female students (6). However the present study revealed no difference between both genders with regard to social support and nutrition behavior, whereas a previous study showed that female were more confident with regards to the social support dimension (6). As said before, this study was the first investigation regarding promoting behaviors among adolescents living in Bandar Abbas. However, there were some limitations in this study. First, in this study, data were collected through self-reported measures, so the results might be biased by survey takers’ understating regarding the degree of healthy behavior and its determinants. However, this limitation is not unique to our study, as the majority of studies conducted on healthy behavior have been based on self-reported data (2, 6, 12, 14, 18). Furthermore, Cronbach´s Alphas of the instrument are borderlines and are somewhat low related to the number of items that might impact the real data. However, this study could be the basis for future studies without these limitations. The cross-sectional study design was the other limitation. Therefore, designing studies that will assess the causes of risky behaviors is strongly recommended. Furthermore, future studies should consider these limitations to better understand the predictors of risky behavior. This study indicated that sedentary life style and physical inactivity is a common and serious problem among the high school students in Bandar Abbas.
